# Teachers’ Emotions and Self-Efficacy: A Test of Reciprocal Relations

**DOI:** 10.3389/fpsyg.2020.01650

**Published:** 2020-08-28

**Authors:** Irena Burić, Ana Slišković, Izabela Sorić

**Affiliations:** Department of Psychology, University of Zadar, Zadar, Croatia

**Keywords:** teachers, emotions, self-efficacy, reciprocal relations, longitudinal design

## Abstract

Previous research has suggested that higher levels of teachers’ self-efficacy (TSE) tend to be positively related to positive teachers’ emotions (e.g., joy, pride) and negatively to negative teachers” emotions (e.g., anger, anxiety). However, these studies predominately relied on cross-sectional design and therefore were unable to test the reciprocal relations between the two constructs. Based on the propositions of social-cognitive theory (Bandura, 1997), TSE may be viewed as an antecedent or as a consequence of emotions. More specifically, TSE may shape emotions since it directs teachers’ attentional, appraisal, and regulatory processes, while emotions may shape TSE since they act as a source of information about teachers’ performance in a given task (i.e., emotions can serve as a filter that determines which efficacy information is seen as salient and how it is interpreted). To test these assumptions, an initial sample of 3010 Croatian teachers (82% female) participated in a longitudinal study based on a full panel design with three measurement points and time lags of approximately 6 months. Teachers taught at different educational levels (i.e., elementary, middle, and secondary schools) and had on average 15.30 years (*SD* = 10.50) of teaching experience. They completed self-report measures that assessed their self-efficacy beliefs and six discrete emotions experienced in relation to teaching and students – joy, pride, love, anger, hopelessness, and exhaustion. An autoregressive cross-lagged analysis showed that teachers’ emotions and TSE are indeed related to each other. However, the direction of this association is not bidirectional as was suggested by theoretical assumptions; instead, it is asymmetrical – higher levels of TSE beliefs predicted higher levels of positive emotions of joy and pride, while higher levels of teachers’ negative emotions of anger, exhaustion, and hopelessness predicted lower levels of teachers’ self-efficacy beliefs.

## Introduction

Teachers experience a variety of discrete emotions of varying intensity while teaching and interacting with students (Sutton and Wheatley, 2003; Meyer and Turner, 2007; Schutz et al., 2007; Sutton, 2007; Spilt et al., 2011; Frenzel, 2014; Burić et al., 2018). These emotions are related to teachers’ instructional practices and their relationships with students, as well as to students’ learning outcomes (Sutton and Wheatley, 2003; Weiner, 2007; Frenzel et al., 2009; Wentzel, 2009; Frenzel, 2014; Hagenauer and Volet, 2014). Moreover, teachers’ emotions contribute to their professional well-being since they may shape burnout levels and job satisfaction, or influence the decision to leave the teaching profession (Macdonald, 1999; Meyer and Turner, 2007; Schutz et al., 2007; Chang, 2009, 2013; Frenzel et al., 2009). Finally, teachers’ emotions seem to be closely related to teacher motivational aspects such as work engagement (Halbesleben, 2010; Burić and Macuka, 2018) or self-efficacy beliefs (Frenzel et al., 2016; Burić et al., 2018).

Teacher self-efficacy (TSE), that is, a teacher’s level of confidence in executing a variety of profession-related activities such as influencing student learning and managing the learning environment (Tschannen-Moran and Hoy, 2001), is one of the most salient motivational characteristics that affect classroom processes and student academic adjustment (e.g., Caprara et al., 2006; Holzberger et al., 2013; Klassen and Tze, 2014; Lauermann and König, 2016; Zee and Koomen, 2016; Burić and Kim, 2020). In addition, TSE has been extensively researched in relation to different aspects of teachers’ well-being such as burnout, stress and coping, job satisfaction, and professional commitment (e.g., Caprara et al., 2006; Moè et al., 2010; Klassen and Chiu, 2011; Zee and Koomen, 2016; Skaalvik and Skaalvik, 2017; Kim and Burić, 2019).

Regardless the existing research on the role of teachers’ emotions and TSE in explaining their instructional practices, students’ outcomes, and diverse well-being indicators, the nature of the relationship between teachers’ emotions and TSE has been rarely studied. Several studies showed that higher levels of TSE are positively related to teachers’ positive emotions (e.g., joy, pride) and negatively to teachers’ negative emotions (e.g., anger, anxiety; Borrachero et al., 2013; Frenzel et al., 2016; Pitkäniemi, 2017; Burić et al., 2018; Burić and Frenzel, 2019). In addition, TSE was found to predict preservice teachers’ practicum performance positively via positive emotions (e.g., love and joy) and negatively via negative emotions (e.g., fear, sadness, and anger; Chen, 2019a). However, since these studies were based on cross-sectional design, the nature and the directionality of the relationship between teachers’ emotions and TSE have remained unknown. Understanding whether TSE causes teachers’ emotions or teachers’ emotions influence TSE may be the first step in effective policy development and intervention implementation that could enhance teachers’ emotional well-being and/or motivation. Therefore, the aim of the present research was to examine the directionality of the association between TSE and a set of discrete emotions (i.e., joy, pride, love, anger, exhaustion, and hopelessness) that teachers experience while teaching and interacting with students.

### Teachers’ Emotions

In recent years, emotions have been recognized as integral parts of teachers’ professional lives. Teachers’ emotions are related to students and their learning, teachers themselves and teaching, as well as to contextual factors (e.g., collegial relationships, principal support, parent’s expectations, educational policies; Wu and Chen, 2018). Even though teachers’ emotions may arise from factors at school (e.g., colleagues and administration), the community (e.g., parents) and from a societal level (e.g., culture and politics), emotions that stem from teaching and interacting with students are the most frequent and intense ones (Chen, 2019b). Teachers rather frequently experience a wide variety of discrete emotions while teaching and interacting with students such as joy, satisfaction, pride, love, anger, exhaustion, hopelessness, anxiety, shame, or boredom (Sutton and Wheatley, 2003; Frenzel et al., 2016; Burić et al., 2018; Chen, 2019b). Such emotions are evoked by a variety of classroom situations and events. For instance, students’ violation of classroom rules or disrespectful behavior toward other students may trigger anger in teachers (Burić and Frenzel, 2019). In contrast, when students strive and succeed academically, teachers may experience joy or pride (Burić et al., 2018). These two examples clearly illustrate that teaching activities and interactions with students are strong sources of teachers’ emotions. Therefore, understanding the causes and triggers of teachers’ emotions, but also their consequences, is of great importance for optimal teachers’ functioning in the classrooms.

The reciprocal model on causes and effects of teacher emotions (Frenzel, 2014) offers a useful theoretical framework for investigating the antecedents and effects of teachers’ emotions. According to this model, teachers hold multiple classroom goals (i.e., to develop students’ subject-specific and socio-emotional competences, to motivate students, and to establish well-functioning relationships with students) whose attainment is evaluated through teachers’ perceptions of students’ behaviors in classroom. Specifically, based on observation of students’ behaviors, teachers appraise whether they accomplished their goals, whether students’ behavior helped them in reaching their goals, and whether they felt competent and capable of achieving their goals. In addition, teachers evaluate who is responsible for attaining (or not attaining) classroom goals as well as how important these goals are. Depending on the content of these cognitive appraisals, different teachers’ emotions may emerge. For instance, for teachers whose goal is to motivate students to learn a certain material by implementing a new teaching method, observation of their students as uninterested and uncooperative could lead to the appraisal of poor coping potential due to insufficient teaching experience and evoke feelings of hopelessness. In contrast, teachers who observe that students make progress and are highly engaged in learning when the material is presented through a new teaching method, may evaluate that their goal is accomplished and consequently experience enjoyment. Finally, according to the model, emotions that result from such cognitive appraisals shape different aspects of teachers’ instructional behavior, that is, cognitive and motivational stimulation, classroom management, and social support (Frenzel, 2014).

Even though the reciprocal model of causes and effects of teacher emotions does not explicitly emphasize the role of TSE in shaping cognitive appraisals and consequently teachers’ emotions, it can be assumed that teachers with higher levels of self-efficacy would have more positive evaluation of their coping potential since they may evaluate themselves as more capable of attaining and optimizing their classroom goals. Such greater coping potential may contribute to the experience of positive teachers’ emotions such as joy or pride. Conversely, teachers with low levels of self-efficacy may appraise their potential to cope with obstacles while attaining and optimizing their classroom goals as poorer, which may lead to the experience of negative emotions such as anger or anxiety.

### Teacher Self-Efficacy

Self-efficacy can be generally defined as a belief about “one’s capability to accomplish a given level of performance” (Bandura, 1986, p. 391). Self-efficacy beliefs influence people’s functioning by shaping their outcome expectations and causal attributions of successes and failures, their motivation to persist even when faced with obstacles, their coping capabilities and emotion regulation mechanisms, as well as their life choices (Bandura, 2012). In the domain of teaching, self-efficacy is best understood as teachers’ beliefs in their capabilities to teach their subject matter, manage the classroom effectively, and motivate and engage students to learn even when this task is difficult (Tschannen-Moran and Hoy, 2001).

According to the model of teachers’ efficacy beliefs (Tschannen-Moran et al., 1998; Hoy et al., 2009), teachers’ efficacy judgments emerge as an interaction between the evaluation of factors that make a specific teaching task easy or difficult to accomplish and the self-evaluation of personal teaching capabilities and limitations that are relevant for successful accomplishment of the task. The resulting self-efficacy beliefs shape the goals teachers set for themselves and their level of aspiration, determine the effort they will invest in reaching these goals as well as the persistence in reaching these goals even when confronted with obstacles and setbacks (Tschannen-Moran and Hoy, 2001; Hoy et al., 2009). Rooted in social-cognitive theory (Bandura, 1997), the model of teachers’ self-efficacy (TSE) beliefs further stipulates that teachers form their self-efficacy beliefs by interpreting information that stems from four sources – mastery experience, vicarious experience, verbal persuasion, and physiological and affective states. Teachers’ mastery experiences are generated in an actual classroom by providing genuine evidence toward whether teachers failed or succeed in a specific task and, therefore, directly influence on TSE. Vicarious experiences may be acquired through observing credible models such as mentors that may be of particular relevance for preservice and novice teachers in forming their self-efficacy beliefs (e.g., Posnanski, 2002; Rice and Roychoudhury, 2003). Mentors, but also colleagues or students, may act as a source of verbal and social persuasion, which may occasionally boost TSE. Lastly, an interpretation of physiological and affective states (i.e., feelings of excitement or anxiety) that accompany different teaching tasks, serves as an information about mastery or incompetence and, thus, contribute to TSE levels (Hoy et al., 2009). The assumptions regarding the sources of information that are relevant for shaping TSE were empirically confirmed in a study on samples of preservice teachers (Pfitzner-Eden, 2016).

Even though mastery experiences are considered the strongest source of self-efficacy beliefs (Bandura, 1997; Hoy et al., 2009; Pfitzner-Eden, 2016), physiological and affective states may also serve an important role in forming teachers’ judgments and confidence. For instance, if teachers feel nervous and stressed while trying to keep their students quiet and focused on learning, they may interpret such physiological and emotional states as indicators of their failure to manage the classroom effectively, which consequently may lower their confidence and sense of efficacy. In contrast, teachers who experience excitement while observing their students who enthusiastically approach and solve even the challenging tasks, may interpret their excitement as a signal of their teaching mastery, which, in turn, boosts their self-efficacy levels.

### The Nature of the Relationship Between Teachers’ Emotions and Self-Efficacy

Based on the theoretical propositions described above, TSE may be viewed as an antecedent and as a consequence of emotions, thus, TSE and emotions should be reciprocally related to each other. As already noted, physiological and affective states are one of the multiple sources of self-efficacy beliefs (Bandura, 1997; Hoy et al., 2009) implying that emotions experienced while teaching and interacting with students may be important in shaping TSE as well. According to the cognitive priming hypothesis, affective states provide information about one’s performance in a given task, that is, they serve as a filter that determines which efficacy information is seen as salient and how it is interpreted. More specifically, affective states that prime positive or negative self-relevant information exert a mood-congruent influence on self-efficacy beliefs (Kavanagh and Bower, 1985) – negative mood evokes negative thoughts and lowers self-efficacy while positive mood enhances positive thoughts and raises self-efficacy. Effects of induced mood on self-efficacy perceptions have been tested in experimental research – while some studies confirmed such effects (e.g., Kavanagh and Bower, 1985; Forgas et al., 1990; Schutte, 2014; Medrano et al., 2016), other studies failed to replicate them (e.g., Cunningham, 1988; Cervone et al., 1994).

However, TSE can also affect teachers’ emotions. Self-efficacy beliefs influence cognitive, motivational, affective, and decisional processes that shape one’s thoughts, well-being, vulnerability to stress and depression, and life choices (Bandura, 2009). More specifically, self-efficacy beliefs direct attention and construal of environmental demands, but also determine an ability to control and manage the emotions and cope with the environmental demands (Lazarus and Folkman, 1984; Bandura, 1997). People with high self-efficacy beliefs use their personal resources more efficiently, have more positive expectations, and set higher goals; they also use effective problem-solving strategies and are more successful in managing stressors they encounter. In contrast, people with low self-efficacy beliefs are more prone to self-doubts and view themselves as less capable to cope with the environmental demands and challenges, which may lead to the experience of negative emotional states such as anxiety, depression, or helplessness (Luszczynska et al., 2005; Schwarzer and Hallum, 2008; Jerusalem and Schwarzer, 2014). Therefore, it can be assumed that teachers with higher levels of TSE could interpret a given classroom situation as less threatening since they believe that they are capable enough to handle its demands and challenges, which may result in the experience of positive emotions. Conversely, teachers with lower levels of TSE could be more prone to self-doubt and to view themselves as less capable to cope with the environmental demands, which will make them more susceptible to the experience of negative emotions.

Research examining the contribution of teachers’ affective experiences on their self-efficacy beliefs, and vice versa, is quite scarce. A few studies that examined the relationship between burnout and TSE by using a longitudinal design found that burnout dimensions act as antecedents of TSE and that higher burnout levels predict lower TSE levels (Brouwers and Tomic, 2000; Kim and Burić, 2019). Interestingly, burnout levels predicted future TSE levels only weakly and inconsistently. In addition, negative physiological and affective states were found to decrease TSE over time through reduction of mastery experiences (Pfitzner-Eden, 2016). A recent longitudinal study showed that teachers’ positive affect positively predicted TSE levels over time, but not vice versa (Burić and Moè, 2020). These results mainly suggest that teachers’ emotions serve as antecedents of TSE – the experience of positive emotions (e.g., joy, pride) may increase TSE, while the experience of negative emotions (e.g., anger, hopelessness) may decrease TSE. However, research presented in this overview and theoretical assumptions suggest that the opposite direction may also be true – higher levels of TSE may favor the experience of positive emotions, while lower levels of TSE may pre predictive for the experience of negative emotions.

### The Present Study

Both teachers’ emotions and TSE have been recognized as important correlates of teachers’ instructional practices and professional well-being indicators as well as students’ academic outcomes (Frenzel, 2014; Frenzel et al., 2016; Zee and Koomen, 2016; Burić et al., 2018). However, the reciprocal relationship between these two constructs has rarely been under scientific inquiry. As assumed by the social-cognitive theory (Bandura, 1997, 2009) and demonstrated by the previous research (e.g., Schwarzer and Hallum, 2008; Kim and Burić, 2019; Burić and Moè, 2020), TSE may act both as an antecedent and as an outcome of teachers’ emotions. In other words, TSE and teachers’ emotions may be reciprocally related to each other.

The aim of the present research was to test this assumption, that is, to examine the directionality of the presumed association between TSE and emotions that teachers experience while teaching and interacting with students. The existing studies assessed teachers’ affective states in a relatively broad manner, that is, either as burnout (Brouwers and Tomic, 2000; Kim and Burić, 2019) or as a more general affect (Pfitzner-Eden, 2016; Burić and Moè, 2020). Consequently, they have neglected the richness and the diversity of teachers’ discrete emotions. The discrete approach to emotions aims at classifying emotions into a number of discrete categories that can be differentiated based on specific cognitive, behavioral, and physiological responses (Lench et al., 2011) and offers a valuable framework for analyzing distinct effects of teachers’ discrete emotions on various outcomes. A few studies that took the discrete approach to teachers’ emotions (Frenzel et al., 2016; Burić et al., 2018; Burić and Macuka, 2018; Burić and Frenzel, 2019) examined the association between teachers’ emotions and TSE in a single time point and left the directionality of the association between the two constructs to remain unknown. To overcome the limitations from previous studies and to fill the existing gap in the literature, we used a longitudinal full panel data on teachers’ discrete emotions (i.e., joy, pride, love, anger, exhaustion, and hopelessness) and TSE collected at three time points on a large sample of teachers (*N* = 3010). The six discrete emotions were chosen since they were found to be amongst the most frequently experienced and most personally relevant emotions that emerge in relation to teaching and interacting with students (Sutton and Wheatley, 2003; Frenzel et al., 2016; Burić et al., 2018). We hypothesized the following:

H1: Teachers’ discrete emotions and TSE will be associated with each other at the same time point – joy, pride, and love will be positively related to TSE, while anger, exhaustion, and hopelessness will be negatively related to TSE.

H2: Current levels of teachers’ discrete emotions will predict future levels of TSE – higher levels of joy, pride, and love will predict higher levels of TSE, while higher levels of anger, exhaustion, and hopelessness will predict lower levels of TSE.

H3. Current levels of TSE will predict future levels of teachers’ discrete emotions – higher levels of TSE will predict higher levels of joy, pride, and love, while higher levels of TSE will predict higher levels of anger, exhaustion, and hopelessness.

## Materials and Methods

### Participants and Procedure

The ethics board of the authors’ university approved this study that was part of a larger research project on antecedents and effects of teachers’ emotions and emotion regulation. An initial sample of 3010 teachers (82% female) from 135 state schools from various locations in Croatia participated in a longitudinal study based on a full panel design with three measurement occasions. At the first assessment point, teachers were on average 41.75 years old (*SD* = 10.44) and had 15.28 years of teaching experience (*SD* = 10.50). Teachers taught at different educational levels – elementary school level (*N* = 867), middle school level (*N* = 1056), and secondary school level (*N* = 935). The remaining teachers either did not report the educational level at which they taught or taught at both the middle school and secondary school educational levels. The participation in the study was anonymous (i.e., answers of teachers collected at different measurement occasions were matched based on self-generated codes known only to teachers) and voluntary.

At each of the three measurement occasions (i.e., Autumn 2015, Spring 2016, and Autumn 2016), separated by time intervals of approximately 6 months, questionnaires were sent to schools via postal service. School psychologists informed the teachers in their schools about the purpose of the research and distributed the questionnaires to teachers who agreed to participate. After the completion of the questionnaires, school psychologists returned them to the research team via postal service. Of all contacted teachers, approximately 50% of them enrolled in the study at the first measurement point. Of the initial sample, 1525 teachers (50.66%) completed the questionnaires also at the second measurement occasion, and 1072 teachers (35.61%) completed the questionnaires at all three occasions.

Due to the dropout of teachers between adjacent data collection points, an attrition analysis was conducted to examine whether teachers who left the study after the first or the second time point differed in demographic characteristics (i.e., gender, educational level, and teaching experience) or substantive variables (i.e., emotions and TSE) from those who remained in the study through its end. The results of this analysis showed that female teachers were more likely to participate in the study at the second [χ^2^(1) = 11.36, *p* < 0.01] and the third time point [χ^2^(1) = 11.89, *p* < 0.01] when compared to the gender composition at the first time point. In addition, in comparison to teachers from elementary and middle schools, high school teachers were less ready to participate in the study at the third time point than at the first [χ^2^(2) = 40.49, *p* < 0.01] and the second time point [χ^2^(2) = 28.13, *p* < 0.01]. No differences were found between the teachers who dropped out either after the first or the second measurement occasion and those who completed questionnaires at all three time points.

Regarding the substantive variables, teachers who left the study after the first time point had somewhat lower levels of TSE [*t*(2944) = −2.09, *p* = 0.037, *d* = 0.08] and higher levels of joy [*t*(2967) = 4.72, *p* < 0.001, *d* = 0.17] and pride [*t*(2939) = 2.93, *p* = 0.003, *d* = 0.11] at the first measurement occasion. Regarding the differences in substantive variables at the second time point, teachers who left the study after the second measurement occasion did not differ from those who participated in all three data collection points. Even though completers and non-completers differed in TSE, joy, and pride measured at the first time point, these effects were quite small (*d* < 0.20; Cohen, 1988) and most likely emerged because of a great statistical power of the present study (i.e., *N* = 3010). Therefore, in order to handle the missing data, the full information maximum likelihood procedure (FIML; Enders, 2010) – which is considered as an appropriate method to handle the missing data in longitudinal studies (Jeličič et al., 2009) – was used.

### Instruments

Teachers’ emotions were measured by the *Teacher Emotion Questionnaire* (TEQ; Burić et al., 2018). The TEQ consisted of six scales measuring six discrete emotions that teachers experience while teaching and interacting with students: *joy* (*n* = 5; example item: “I am joyful when the class atmosphere is positive”), *pride* (*n* = 6; example item: “I am filled with pride when I make a student interested in my subject”), *love* (*n* = 6; example item: “I feel warmth when I just think about my students”), *anger* (*n* = 5; example item: “Some students make me so angry that my face goes red”), *exhaustion* (*n* = 7; example item: “When I finish my work, I feel drained”), and *hopelessness* (*n* = 6; example item: “It seems to me that I cannot do anything to get through to some students”). Teachers rated all items on a five-point scale ranging from 1 (strongly disagree) to 5 (strongly agree). For the full list of items, please see the Appendix.

TSE was assessed by the *Teacher Self-Efficacy Scale* (TSES; Schwarzer et al., 1999). The TSES consisted of 10 items measuring teachers’ sense of efficacy in relation to their tasks’ accomplishment, skill development, and interactions with students, parents, and colleagues, as well as to coping with job stress. An example item is: “Even if I get disrupted while teaching, I am confident that I can maintain my composure and continue to teach well.” Teachers rated the items on a four-point scale ranging from 1 (not at all true) to 4 (exactly true).

The internal consistency coefficients (i.e., Cronbach α’s) for all scales are presented in Tables 1, 2.

**TABLE 1:** Descriptive statistics, reliability coefficients and correlations for positive emotions.

	Variable	1	2	3	4	5	6	7	8	9	10	11	12	13	14
1	Gender	–	0.05**	0.14**	0.15**	0.16**	0.13**	0.09**	0.11**	0.17**	0.14**	0.15**	0.02	0.02	–0.01
2	Experience		–	−0.07*	–0.01	0.06	0.03	0.09**	0.09**	0.13**	0.15**	0.16**	0.02	0.07**	0.03
3	Joy T1			–	0.48**	0.42**	0.63**	0.39**	0.35**	0.43**	0.26**	0.28**	0.29**	0.18**	0.19**
4	Joy T2				–	0.47**	0.42**	0.64**	0.38**	0.33**	0.44**	0.30**	0.25**	0.28**	0.20**
5	Joy T3					–	0.41**	0.44**	0.65**	0.35**	0.37**	0.48**	0.24**	0.22**	0.29**
6	Pride T1	–					–	0.59**	0.57**	0.60**	0.40**	0.40**	0.36**	0.27**	0.28**
7	Pride T2							–	0.62**	0.49**	0.65**	0.48**	0.32**	0.39**	0.30**
8	Pride T3								–	0.43**	0.48**	0.66**	0.29**	0.28**	0.29**
9	Love T1									–	0.72**	0.66**	0.37**	0.29**	0.28**
10	Love T2										–	0.73**	0.31**	0.34**	0.30**
11	Love T3											–	0.29**	0.25**	0.36**
12	TSE T1												–	0.57**	0.62**
13	TSE T2													–	0.61**
14	TSE T3														–
15	M	–	15.28	4.73	4.72	4.74	4.43	4.37	4.38	4.08	3.94	3.95	3.37	3.33	3.29
16	SD	–	10.50	0.38	0.40	0.38	0.51	0.54	0.54	0.64	0.69	0.71	0.40	0.41	0.44
17	Cronbach α	–	–	0.85	0.87	0.87	0.86	0.86	0.87	0.87	0.90	0.90	0.84	0.86	0.88

*p < 0.05, **p < 0.01; T1, T2, and T3 = time points; TSE = Teacher Self-Efficacy.

**TABLE 2:** Descriptive statistics, reliability coefficients and correlations for negative emotions.

	Variable	1	2	3	4	5	6	7	8	9	10	11	12	13	14
1	Gender	–	0.05**	0.06**	0.05	0.01	0.09**	0.10**	0.10**	0.08**	0.05*	0.02	0.02	0.02	–0.01
2	Experience		–	0.06**	0.02	–0.01	0.06**	0.04	–0.02	–0.02	−0.06**	–0.06	–0.02	0.07**	0.03
3	Anger T1			–	0.65**	0.60**	0.65**	0.47**	0.46**	0.74**	0.55**	0.50**	−0.35**	−0.30**	−0.36**
4	Anger T2				–	0.68**	0.53**	0.69**	0.53**	0.53**	0.75**	0.58**	−0.29**	−0.38**	−0.36**
5	Anger T3					–	0.48**	0.54**	0.68**	0.50**	0.59**	0.78**	−0.29**	−0.30**	−0.39**
6	Exhaustion T1						–	0.68**	0.63**	0.62**	0.45**	0.44**	−0.26**	−0.22**	−0.31**
7	Exhaustion T2							–	0.68**	0.45**	0.60**	0.50**	−0.22**	−0.30**	−0.29**
8	Exhaustion T3								–	0.60**	0.45**	0.63**	−0.23**	−0.23**	−0.36**
9	Hopelessness T1									–	0.64**	0.58**	−0.39**	−0.31**	−0.37**
10	Hopelessness T2										–	0.65**	−0.36**	−0.46**	−0.41**
11	Hopelessness T3											–	−0.38**	−0.34**	−0.48**
12	TSE T1												–	0.57**	0.62**
13	TSE T2													–	0.61**
14	TSE T3														–
15	M	–	15.28	2.31	2.33	2.34	2.87	2.84	2.88	3.07	2.58	2.56	3.37	3.33	3.29
16	SD	–	10.50	0.73	0.75	0.77	0.88	0.85	0.86	0.84	0.76	0.78	0.40	0.41	0.44
17	Cronbach α	–	–	0.79	0.81	0.82	0.91	0.92	0.92	0.86	0.88	0.89	0.84	0.86	0.88

T1, T2, and T3, time points; TSE, Teacher Self-Efficacy. *p < 0.05, **p < 0.01.

### Data Analysis

**FIGURE 1:**
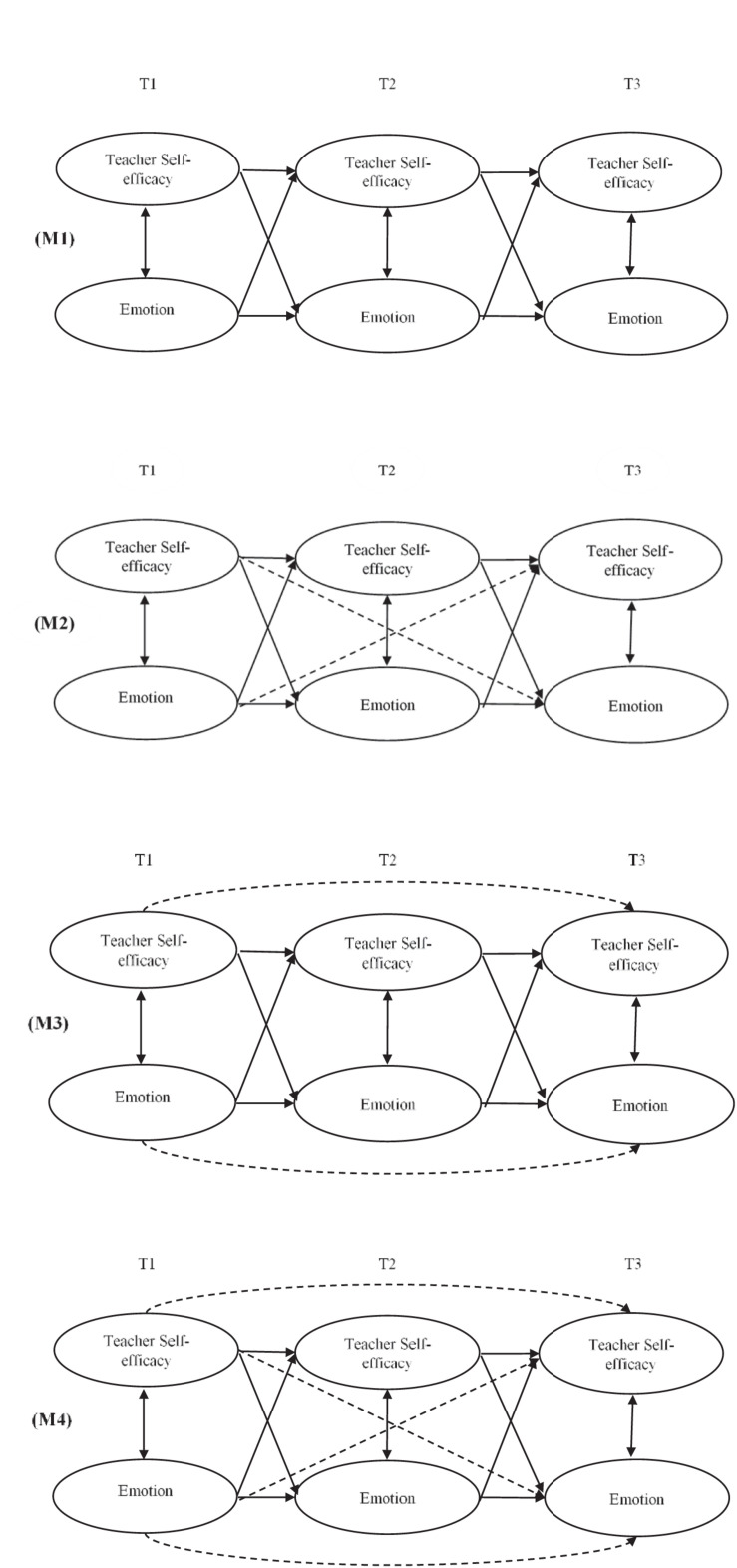
Structural models.

Data were analyzed in three steps. *First*, the Pearson correlation coefficients between the substantive variables (i.e., emotions and TSE) and teachers’ demographics (i.e., gender, years of teaching experience) were calculated. *Second*, to ensure that the measurement of each of the constructs across time points had not changed, the measurement invariance for each of the six emotions and TSE across time was tested. It was suggested that configural invariance (i.e., the invariance of configuration of the relationships between the latent construct and its indicators across time points) and metric invariance (i.e., the invariance of factor loadings across time points) should be established prior to testing the structural relationships between the constructs (Byrne, 2012). While testing the measurement invariance, scale items were used as indicators of each of the latent constructs (i.e., six emotions and TSE). In addition, to control for systematic measurement error, the autocorrelations of the items’ residuals across time points were specified (Marsh and Hau, 1996). *Third*, to test the hypothesized structural relationships between teachers’ emotions and TSE, four structural models were specified, tested, and compared to each other: (1) a model specifying only first order autoregressive and cross-lagged paths (M1); (2) a model specifying first order autoregressive and first- and higher order cross-lagged paths (M2); (3) a model specifying first- and higher order autoregressive paths and first-order cross-lagged paths (M3); and (4) a model specifying both first- and higher order autoregressive paths and first- and higher order cross-lagged paths. The set of the four models was tested for each of the six emotions separately in order to reduce model complexity and avoid potential problems with multicollinearity – in total, 24 structural models were tested. In each of these models, a particular emotion and TSE were allowed to correlate within a single time point. The tested models are shown in Figure 1.

The analyses were conducted using Mplus 8.0 (Muthén and Muthén, 1998–2017). The maximum-likelihood estimation method was used to estimate model parameters. The quality of model fit was evaluated based on several criteria: comparative fit index (CFI), Tucker-Lewis index (TLI), root-mean-square error of approximation (RMSEA), and standardized root-mean residual (SRMR). Values of CFI and TLI that are above 0.90 and 0.95 are indicative of acceptable and excellent fit, respectively (Hu and Bentler, 1999). Values of RMSEA lower than 0.06 and values of SRMR lower than 0.08, indicate good fit (Browne and Cudeck, 1992). To determine the better fitting model when testing competing measurement and structural models, a chi-square difference test (Δχ^2^) was calculated. However, χ^2^ values tend to be significant when calculated on data from large sample sizes (as was the case in this study too), thus leading to overly high model rejection rates (Marsh et al., 1988). Thus, for the evaluation of measurement models, ΔCFI ≤ 0.01 and ΔRMSEA ≥ 0.015 criteria were additionally used – models with lower ΔCFI and ΔRMSEA values should be preferred (Cheung and Rensvold, 2002; Chen, 2007).

## Results

### Correlations

Descriptive statistics and Pearson correlation coefficients are shown in Tables 1, 2. As can be seen, positive teachers’ emotions of joy, love, and pride positively correlated with TSE within the same time point and across time. In contrast, teachers’ negative emotions of anger, exhaustion, and hopelessness correlated negatively with TSE within the single time point and across time. Concerning teachers’ demographic variables, female teachers reported somewhat higher levels of all emotions (except of anger assessed at Time 2 and Time 3), while more experienced teachers reported higher levels of love, pride, anger, exhaustion, and TSE, and lower levels of joy and hopelessness. However, even though statistically significant due to a large sample size, these correlations were quite low and inconsistent across time.

### Measurement Invariance

The results of the comparison of configural invariance model with more restrictive metric invariance model for each of the six emotions are shown in Table 3. The results of series of Δχ^2^ tests suggest that imposing restrictions of equal factor loadings across time did not change the overall fit of the models for love [Δχ^2^(28) = 36.32, *p* > 0.05], exhaustion [Δχ^2^(30) = 42.49, *p* > 0.05], and hopelessness [Δχ^2^(28) = 38.91, *p* > 0.05]. However, when compared to the configural invariance models, the metric invariance models of joy, pride, and anger had somewhat lower fit: Δχ^2^(26) = 39.44, *p* < 0.05, *p* > 0.01; Δχ^2^(28) = 51.69, *p* < 0.05, *p* > 0.01; and Δχ^2^(26) = 43.37, *p* < 0.01, *p* > 0.05, respectively. However, as already noted, Δχ^2^ tends to overly reject even the models with satisfactory fit due to its sensitivity to a large sample size. Indeed, the ΔCFI and ΔRMSEA values were well below the recommended threshold in each of the six model comparisons – joy (ΔCFI = 0.001, ΔRMSEA = 0.00), pride (ΔCFI = 0.001, ΔRMSEA = 0.00), love (ΔCFI = 0.00, ΔRMSEA = 0.00), anger (ΔCFI = 0.001, ΔRMSEA = 0.001), exhaustion (ΔCFI = 0.00, ΔRMSEA = 0.00), and hopelessness (ΔCFI = 0.001, ΔRMSEA = 0.00). Therefore, a sufficient amount of metric invariance (i.e., equal factor loadings) across time was achieved.

**TABLE 3:** Fit statistics of tested models.

Model Type	χ^2^ (df)	CFI	TLI	RMSEA (90% CI)	SRMR
**Joy–TSE**					
Configural invariance	2522.05 (879)	0.953	0.947	0.025 (0.024, 0.026)	0.038
Metric invariance	2561.49 (905)	0.952	0.948	0.025 (0.024, 0.026)	0.042
M1	2713.77 (909)	0.948	0.943	0.026 (0.025, 0.027)	0.052
M2	2711.57 (907)	0.948	0.943	0.026 (0.025, 0.027)	0.051
M3	2562.15 (907)	0.952	0.948	0.025 (0.024, 0.026)	0.042
M4	2561.49 (905)	0.952	0.948	0.025 (0.024, 0.026)	0.042
**Pride–TSE**					
Configural invariance	2622.83 (1011)	0.957	0.952	0.023 (0.022, 0.024)	0.037
Metric invariance	2674.52 (1039)	0.956	0.953	0.023 (0.022, 0.024)	0.040
M1	2844.31 (1043)	0.952	0.948	0.024 (0.023, 0.025)	0.049
M2	2841.05 (1041)	0.952	0.948	0.024 (0.023, 0.025)	0.048
M3	2674.54 (1041)	0.956	0.953	0.023 (0.022, 0.024)	0.040
M4	2674.52 (1039)	0.956	0.953	0.023 (0.022, 0.024)	0.040
**Love–TSE**					
Configural invariance	2761.38 (1011)	0.959	0.954	0.024 (0.023, 0.025)	0.041
Metric invariance	2797.70 (1039)	0.959	0.955	0.024 (0.023, 0.025)	0.043
M1	2948.42 (1043)	0.955	0.952	0.025 (0.024, 0.026)	0.050
M2	2946.54 (1041)	0.955	0.952	0.025 (0.024, 0.026)	0.049
M3	2799.33 (1041)	0.959	0.955	0.024 (0.023, 0.025)	0.043
M4	2797.70 (1039)	0.959	0.955	0.024 (0.023, 0.025)	0.043
**Anger–TSE**					
Configural invariance	2482.05 (879)	0.951	0.944	0.025 (0.023, 0.026)	0.038
Metric invariance	2525.42 (905)	0.950	0.945	0.024 (0.023, 0.026)	0.040
M1	2649.02 (909)	0.946	0.942	0.025 (0.024, 0.026)	0.047
M2	2630.81 (907)	0.947	0.942	0.025 (0.024, 0.026)	0.045
M3	2527.28 (907)	0.950	0.946	0.024 (0.023, 0.026)	0.040
M4	2525.42 (905)	0.950	0.945	0.024 (0.023, 0.026)	0.040
**Exhaustion–TSE**					
Configural invariance	2855.62 (1152)	0.964	0.960	0.022 (0.021, 0.023)	0.036
Metric invariance	2898.11 (1182)	0.964	0.961	0.022 (0.021, 0.023)	0.037
M1	3058.42 (1186)	0.961	0.958	0.023 (0.022, 0.024)	0.045
M2	3048.11 (1184)	0.961	0.958	0.023 (0.022, 0.024)	0.044
M3	2903.98 (1184)	0.964	0.961	0.022 (0.021, 0.023)	0.037
M4	2898.11 (1182)	0.964	0.961	0.022 (0.021, 0.023)	0.037
**Hopelessness–TSE**					
Configural invariance	2532.59 (1011)	0.960	0.955	0.022 (0.021, 0.023)	0.033
Metric invariance	2571.50 (1039)	0.959	0.956	0.022 (0.021, 0.023)	0.034
M1	2709.39 (1043)	0.956	0.952	0.023 (0.022, 0.024)	0.043
M2	2691.49 (1041)	0.956	0.953	0.023 (0.022, 0.024)	0.040
M3	2581.30 (1041)	0.959	0.956	0.022 (0.021, 0.023)	0.035
M4	2571.50 (1039)	0.959	0.956	0.022 (0.021, 0.023)	0.034

*M1, model with the first order stability and cross-lagged paths; M2, model with the first order stability and the first- and higher order cross-lagged paths; M3, model with the first- and higher order stability paths and the first order cross-lagged paths; M4, model with the first- and higher order stability and cross-lagged paths.*

### Structural Models

The results of the test of the four specified structural models for each of the six emotions are shown in Table 3 while the results of χ^2^ difference tests used to compare the competing structural models are presented in Table 4. In all models except the Hopelessness–TSE model, model M3 with specified the first- and higher order autoregressive paths but only the first order cross-lagged paths had better fit than models M1 (i.e., only the first autoregressive and cross-lagged paths) and M2 (i.e., first order autoregressive paths but also the first- and higher order cross-lagged paths) and did not differ from more complex model M4 (i.e., both the first- and higher order autoregressive and cross-lagged paths). These results indicate that model M3 (i.e., which specified the first- and second order autoregressive paths and only first order cross-lagged paths) should be preferred in the case of all emotions except hopelessness. Concerning the Hopelessness–TSE model, model M4 (i.e., which specified both the first- and higher order autoregressive and cross-lagged paths) had better fit than more parsimonious model M3 and was thus chosen as the preferred one.

**TABLE 4:** Results of model comparison based on χ^2^ difference test.

	Joy–TSE	Pride–TSE	Love–TSE	Anger–TSE	Exhaustion–TSE	Hopelessness–TSE
	Δχ^2^ (df)	Δχ^2^ (df)	Δχ^2^ (df)	Δχ^2^ (df)	Δχ^2^ (df)	Δχ^2^ (df)
M1 vs. M2	2.21 (2)	3.25 (2)	1.88 (2)	18.21 (2)*	10.31* (2)	17.90* (2)
M1 vs. M3	151.63** (2)	169.77** (2)	149** (2)	121.74** (2)**	154.44** (2)	128.09** (2)
M2 vs. M3	149.42 (0)	166.51	147.21 (0)	103.53 (0)	114.13 (0)	110.19 (0)
M3 vs. M4	0.66 (2)	0.02 (2)	1.64 (2)	1.93 (2)	5.88 (2)	9.80 (2)*

*p < 0.01, **p < 0.001.

The size and statistical significance of the autoregressive and cross-lagged structural paths of the best fitting models (i.e., M4 for hopelessness and M3 for all other emotions) are presented in Table 5. It was found that TSE at Time 1 positively predicted joy at Time 2, while TSE at Time 2 also positively predicted joy at Time 3. However, current levels of joy failed to predict future levels of TSE. Similarly, TSE measured at Time 1 positively predicted pride measured at Time 2, however, TSE at Time 2 was unrelated to pride at Time 3. Again, current levels of pride were unrelated to future levels of TSE. Surprisingly, current levels of TSE failed to predict future levels of love and current levels of love were unrelated to future levels of TSE.

**TABLE 5:** Stability and cross-lagged paths of the best fitting models.

	Joy	TSE	Pride	TSE	Love	TSE
**Stability paths**						
T1 → T2	0.516**	0.636**	0.612**	0.619**	0.763**	0.616**
T2 → T3	0.328**	0.394**	0.458**	0.384**	0.563**	0.386**
T1 → T3	0.293**	0.414**	0.331**	0.418**	0.289**	0.414**
**Cross-lagged paths**						
T1 → T2	0.117**	0.009	0.108**	0.043	0.007	0.053
T2 → T3	0.106**	0.019	0.040	0.027	–0.043	0.027

	**Anger**	**TSE**	**Exhaustion**	**TSE**	**Hopelessness**	**TSE**

**Stability paths**						
T1 → T2	0.717**	0.589**	0.719**	0.618**	0.656**	0.596**
T2 → T3	0.593**	0.343**	0.527**	0.367**	0.516**	0.360**
T1 → T3	0.230**	0.398**	0.261**	0.413**	0.245**	0.388**
**Cross-lagged paths**						
T1 → T3	–0.047	−0.108**	–0.035	−0.070**	−0.123**	−0.091**
T2 → T3	–0.004	−0.144**	–0.027	−0.097**	0.040	0.051
T1 → T3^†^	–	–	–	–	−0.109**	–0.063

^†^Model Hopelessness–TSE included both the higher-order stability and cross-lagged paths. **p < 0.01.

Concerning negative emotions, the results were quite different. Anger at Time 1 negatively predicted TSE at Time 2, while anger assessed at the second at Time 2 negatively predicted TSE at Time 3. However, current levels of TSE failed to predict future levels of anger. Similar regression coefficients were found in the model with exhaustion; exhaustion measured at Time 1 negatively predicted TSE at Time 2, while exhaustion measured at Time 2 predicted TSE at Time 3. Again, the opposite direction of association was not established – current levels of TSE did not predict future levels of exhaustion. Interestingly, TSE measured at Time 1 negatively predicted hopelessness measured at Time 2 and Time 3. In addition, hopelessness measured at Time 1 negatively predicted TSE measured at Time 2, but not TSE measured at Time 3. However, TSE at Time 2 failed to predict hopelessness at Time 3. The opposite was also true – hopelessness at Time 2 was unrelated to TSE at Time 3.

Regarding the relationship between TSE and emotions within the same time point, joy correlated positively with TSE within each measurement occasion (*r* = 0.343, *p* < 0.01; *r* = 0.201, *p* < 0.01; and *r* = 0.182, *p* < 0.01 at Time 1, Time 2, and Time 3, respectively). The same was true for pride (*r* = 0.431, *p* < 0.01; *r* = 0.301, *p* < 0.01; and *r* = 0.348, *p* < 0.01 at Time 1, Time 2, and Time 3, respectively), and love (*r* = 0.425, *p* < 0.01; *r* = 0.248, *p* < 0.01; and *r* = 0.343, *p* < 0.01 at Time 1, Time 2, and Time 3, respectively). Correlations between TSE and negative emotions within each time point were negative – TSE was negatively related to anger (*r* = −0.443, *p* < 0.01; *r* = −0.318, *p* < 0.01; and *r* = −0.232, *p* < 0.01 at Time 1, Time 2, and Time 3, respectively), exhaustion (*r* = −0.294, *p* < 0.01; *r* = −0.231, *p* < 0.01; and *r* = −0.252, *p* < 0.01 at Time 1, Time 2, and Time 3, respectively), and hopelessness (*r* = −0.471, *p* < 0.01; *r* = −0.407, *p* < 0.01; and *r* = −0.352, *p* < 0.01 at Time 1, Time 2, and Time 3, respectively).

In sum, the obtained results confirmed the first hypothesis – within the same time point, positive teachers’ emotions of joy, pride, and love correlated positively with TSE, while negative teachers’ emotions of anger, exhaustion, and hopelessness correlated negatively with TSE (H1). The second hypothesis was only partially confirmed – higher current levels of negative emotions of anger, exhaustion, and hopelessness negatively predicted future levels of TSE. However, the same direction of association was not found in models with positive emotions (H2). In contrast, current TSE levels positively predicted future levels of joy and pride, and negatively predicted future levels of hopelessness. Nonetheless, the importance of TSE in predicting these emotions was not consistent between the adjacent time points. Therefore, the third hypothesis was only partially confirmed (H3). Lastly and contrary to expectations, love failed to predict TSE and vice versa – current levels of TSE were unrelated to future levels of emotions.

## Discussion

The aim of the current study was to examine whether teachers’ discrete emotions (i.e., joy, pride, love, anger, exhaustion, and hopelessness) and TSE are reciprocally related to each other. In reaching this aim, a three-wave longitudinal panel design on a large sample of teachers was implemented. In spite of the growing research interest in teachers’ emotions and abundance of research on TSE, the relationship between these two constructs, as well as its direction, have rarely been in the focus of researchers. Therefore, the results of this research may deepen our understanding of the interplay of teachers’ emotions and TSE, which is considered as one of the most important beliefs in teachers’ motivation literature.

As expected, teachers’ emotions and TSE were concurrently associated with each other at each of the three time points – teachers who reported an experience of higher levels of positive emotions of joy, pride, also reported higher levels of TSE. The opposite pattern of association was found for negative emotions – teachers who had higher levels of anger, exhaustion, and hopelessness also had lower levels of TSE. These results are consistent with previous cross-sectional studies that found positive correlation between teachers’ positive discrete emotions (i.e., enjoyment, pride) and TSE, and negative correlation between negative discrete emotions (i.e., anger, anxiety, hopelessness) and TSE (Frenzel et al., 2016; Burić et al., 2018; Burić and Frenzel, 2019).

Even though related concurrently, the reciprocal relationship between emotions and TSE was not consistent across different discrete emotions and time. As stipulated by the second hypothesis, current levels of teachers’ emotions should predict future levels of TSE. According to the social-cognitive theory (Bandura, 1997) and the model of teachers’ efficacy beliefs (Tschannen-Moran et al., 1998; Hoy et al., 2009), physiological and affective states may act as a strong source of information in forming TSE. For instance, teachers who feel frustrated, nervous, or exhausted during teaching may interpret these feelings as a sign of incompetence, which may reduce their TSE levels. In contrast, feelings of excitement and contentment may serve as a signal that the class has been carried out efficiently, which boosts teachers’ confidence levels and a sense of mastery and, consequently, enhance TSE.

The obtained results showed that these assumptions are true only concerning negative emotions – teachers who reported to experience higher levels of anger, exhaustion, and hopelessness at the current time point, also reported lower levels of TSE at subsequent assessment. While this direction of prediction was stable across time for anger and exhaustion, hopelessness measured at the first measurement occasion predicted TSE only and the second measurement occasion (i.e., the path from Time 2 to Time 3 was near zero). In addition, none of the paths from positive emotions to TSE reached statistical significance. Therefore, the second hypothesis was only partially supported. These findings are in line with previous longitudinal studies that showed negative effects of negative affective and physiological states on forming TSE (Pfitzner-Eden, 2016) or negative effects of burnout in predicting TSE (Kim and Burić, 2019). However, the insignificant paths from positive emotions to TSE failed to support previous research that demonstrated a positive contribution of positive affect in shaping TSE over time (Burić and Moè, 2020). Finding only a partial support of the second hypothesis may reflect the fact that the experience of negative emotions while teaching and interacting with students provides a much stronger source of information about teachers’ competence and mastery in a given task when compared to the experience of positive emotions. This explanation fits within the “bad is stronger than good” observation that occurs with regard to emotions as well. More specifically, there is abundance of empirical evidence showing that negative affective experiences have stronger effects on cognitive processing, regulatory mechanisms, and behavior than positive affective experiences (Baumeister et al., 2001).

According to the third hypothesis, current TSE levels should predict future teachers’ emotions. People with high levels of self-efficacy are more confident, set higher goals for themselves, and are more persistent when faced with obstacles (Bandura, 1997). In addition, it was suggested that self-efficacy raises people’s coping potential to handle challenges and overcome obstacles more successfully which reduces the experience of negative emotional states and promotes the experience of positive emotional states (Lazarus and Folkman, 1984; Schwarzer and Hallum, 2008; Skaalvik and Skaalvik, 2017). Again, these propositions were only partially confirmed. As expected, TSE positively predicted joy and pride over time, and negatively hopelessness. Teachers with a greater sense of efficacy provide instruction of higher quality and have greater power in promoting students’ motivational, affective, and cognitive outcomes (Holzberger et al., 2013; Klassen and Tze, 2014; Zee and Koomen, 2016; Burić and Kim, 2020). Consequently, those teachers who are more likely to reach classroom goals they set (i.e., to develop students’ subject-specific and socio-emotional competencies, to motivate students, and to establish positive relationships with students), should also more frequently experience positive emotions, as suggested by the reciprocal model on causes and effects of teacher emotions (Frenzel, 2014). Similarly, high self-efficacy beliefs and consequent better teaching performance should prevent the occurrence of the devastating emotion of hopelessness.

However, current levels of TSE failed to predict future levels of the other two analyzed negative emotions, that is, anger and exhaustion. Anger is an emotion that is typically accompanied by appraisals of other blame (e.g., by students who are being inattentive on purpose) for blocked goals (Berkowitz, 1993) and, thus, may be less affected by teachers’ sense of efficacy. Similarly, teachers’ exhaustion is typically caused by the teaching activity itself that is dynamic and unpredictable and, oftentimes, cognitively and emotionally demanding and draining (Burić et al., 2018). In other words, even teachers with high sense of efficacy may feel exhausted by teaching and intense interactions with students. Lastly, in their review of literature on the effects of TSE on teachers’ well-being, Zee and Koomen (2016) concluded TSE may be of higher predictive value for positive outcomes (i.e., personal accomplishment) than for negative ones (i.e., stress and burnout), implying that high TSE levels help teachers to stay motivated and satisfied.

Surprisingly, teachers’ love failed to predict TSE and TSE failed to predict love. Failure to find any association between love and TSE longitudinally can be explained by a universality of feelings of love and caring in the teaching profession (e.g., Isenbarger and Zembylas, 2006). Caring and feelings of love and affection toward children and students are inherent to teaching and may be less affected or caused by TSE which is mostly concerned with teachers’ evaluation of their capabilities to provide high quality classroom practices, to efficiently manage the classroom, or to engage students in learning (Hoy et al., 2009). Lastly, the true reciprocal relationship was established only for hopelessness – TSE assessed at the first measurement occasion predicted hopelessness at the second measurement occasion and vice versa. This finding may be partly explained by the content of items of hopelessness scale that most closely resemble low self-efficacy beliefs which is also reflected in moderately high correlations between TSE and hopelessness within and across time points.

### Limitations and Directions for Future Research

The present research has several limitations that should be taken into consideration when interpreting the results. First, even though the measurement instrument used to assess teachers’ emotions was proven to be reliable and valid (i.e., its scales showed good internal consistency and theoretically meaningful relations with external variables such as TSE, positive and negative affective experiences, emotional labor, job satisfaction, work engagement, etc.) across studies (Burić and Macuka, 2018; Burić et al., 2018, 2019), items of different scales vary in its specificity and representation of different emotion components. For instance, some of the items of positive emotion scales assess emotions that teachers experience in relation to specific classroom events such as creating positive classroom atmosphere, reaching classroom goals, or making students interested in learning which are also inherent to TSE as well. In contrast, such classroom events are less represented in items of negative emotion scales which makes them less confounded with TSE. This imbalance in the representation of specific classroom events (that are also constituent elements of TSE) across positive and negative emotion scales may have added to the discrepancies of the longitudinal relationship of TSE with positive and negative emotions.

Second, this study took place in the Croatian educational context that has been undergoing transition and change within the European integration processes for the last several years (Cain and Milovic, 2010). Future studies should aim at replicating these findings in different national and/or cultural contexts. Next, the sample of teachers enrolled in the study was convenient. Approximately 50% of all approached teachers agreed to participate. Even though this response rate is higher than in previous studies with teacher population (Metler, 2003), it still raises questions regarding the characteristics of teachers who declined to enroll. Moreover, based on the mean values of the results on substantive variables, it can be seen that participating teachers had moderate or high levels of TSE and positive emotions and moderate to low levels of negative emotions. Such range restrictions may lead to attenuation of the sizes of regression weights and, therefore, to underestimation of true effect sizes. Finally, even though teachers were informed that their answers would be treated with strict confidentiality, the possibility of giving socially desirable responses cannot be excluded. Therefore, future studies may wish to test hypotheses regarding the reciprocal relationship between TSE and emotions after controlling for socially desirable responses.

### Theoretical and Practical Implications

The results of this study suggested that the relationship between teachers’ emotions and TSE is more asymmetrical than bidirectional – while TSE tends to predict positive emotions, negative emotions tend to predict TSE. Taking the discrete approach to emotions and exploring the predictive strength of an array of six positive and negative emotions of different qualities, contribute to the scarce base of knowledge on the role of teachers’ emotional experience in shaping one of the most studied motivational constructs, that is, TSE (Klassen et al., 2011). In addition, findings from the present research clearly demonstrated a beneficial role of TSE in shaping teachers’ emotional well-being by promoting the experience of positive emotions (i.e., joy and pride) and preventing the experience of the devastating and debilitating emotion of hopelessness. Since the majority of studies that aimed at examining the role of TSE in explaining teachers’ well-being were based on a cross-sectional design (Zee and Koomen, 2016), implementing a longitudinal full panel design helps to illuminate the protective role of TSE for teachers’ emotional lives.

The finding of an adverse role of teachers’ negative emotions in shaping TSE may be used in trainings and intervention programs for both in-service and pre-service teachers. More specifically, teachers could be trained to use efficient and adaptive emotion regulation strategies that would hamper or reduce the experience of negative emotions such as anger or exhaustion. For instance, using reappraisal (i.e., modifying the way one thinks either about a situation that evokes an emotion or about one’s capacity to manage it) or attempts to actively modify the features of the situation that evoked an emotion may prove fruitful in preventing teachers’ negative emotional experiences (Gross and John, 2003; Burić et al., 2017). Such regulative attempts can help in preventing the adverse effects of negative emotions while forming judgments about one’s teaching competence. Similarly, fostering TSE beliefs by ensuring opportunities for mastery experience and success as well as providing beginning teachers with competent mentors or senior colleagues who would serve as both models and persuaders, may promote positive aspects of teachers’ emotional well-being. In other words, interventions and training aimed at improving teachers’ emotion regulation abilities may protect TSE, while providing opportunities to build TSE may promote their positive emotional experiences.

## Conclusion

In conclusion, the present research showed that teachers’ emotions and TSE are indeed tightly related to each other. However, the direction of this association is not bidirectional as was suggested by theoretical assumptions; instead it is asymmetrical – it seems that TSE has greater power for enhancing positive emotions, while negative emotions has stronger potential for deteriorating TSE. In addition, the current study suggests that taking the discrete approach to emotions may be more valuable for understanding the role of emotions in shaping TSE than the dimensional approach to emotions (i.e., conceptualizing teachers’ emotional states as two broad affective categories – positive and negative affect; Tellegen et al., 1999; Russell, 1980).

## Data Availability Statement

The datasets generated for this study are available on request to the corresponding author.

## Ethics Statement

The studies involving human participants were reviewed and approved by Ethical Committee of Department of Psychology at University of Zadar. The patients/participants provided their written informed consent to participate in this study.

## Author Contributions

IB contributed to this manuscript by its funding acquisition, conceptualization, data collection, statistical analysis, and writing the original and revised draft of the manuscript. AS and IS contributed by project administration, data collection, and writing the original draft of the manuscript. All authors contributed to the article and approved the submitted version.

## Conflict of Interest

The authors declare that the research was conducted in the absence of any commercial or financial relationships that could be construed as a potential conflict of interest.

## Appendix

### Teacher Emotion Questionnaire Items

#### Joy

I am glad when I achieve teaching goals that are set.

I am joyful when the class atmosphere is positive.

I am happy when I manage to motivate students to learn.

I am happy when students understand the material.

Exerting a positive influence on my students makes me happy.

#### Pride

I feel like a winner when my students succeed.

Due to my students’ achievements, I feel as if I am “growing.”

I am filled with pride when I make a student interested in my subject.

Meetings with successful former students of mine make me proud.

When I am proud of my students, I feel that my confidence is growing.

Pride due to my students’ achievements confirms to me that I am doing a good job.

#### Love

I feel warmth when I just think about my students.

I love my students.

My students evoke feelings of love inside me.

I feel affection toward my students.

I wish to hug my students since I like them so much.

I honestly care about each of my student.

#### Anger

I sweat from frustration when the class is not carried in the way it is supposed to.

The reactions of some students frustrate me so much that I would rather just quit the job.

The frustration I feel while working with students undermines my job motivation.

Some students make me so angry that my face goes red.

I get an anger-caused headache from the behavior of some students.

#### Exhaustion

At the end of my working day, I just want to rest.

When I finish classes, I feel numbed.

My job sometimes makes me so tired that all I want to do is “switch off.”

Due to the speedy pace of work, at the end of the day I feel as if I am going to fall down.

Sometimes I am so exhausted at work that I only think about how to endure.

When I finish my work, I feel drained.

Sometimes working with children makes me so tired that I can barely move.

#### Hopelessness

I feel I cannot do anything more to correct the behavior of some students.

While working with completely unmotivated students, I feel there is no way out.

Because of the behavior of some students, I feel completely helpless.

I feel hopeless when I think about the achievement of some students.

It seems to me that I cannot do anything to get through to some students.

I feel defenseless because I cannot help some of my students.
